# Tomato Brown Rugose Fruit Virus Contributes to Enhanced Pepino Mosaic Virus Titers in Tomato Plants

**DOI:** 10.3390/v12080879

**Published:** 2020-08-11

**Authors:** Chen Klap, Neta Luria, Elisheva Smith, Lior Hadad, Elena Bakelman, Noa Sela, Eduard Belausov, Oded Lachman, Diana Leibman, Aviv Dombrovsky

**Affiliations:** 1 Department of Plant Pathology and Weed Research, Agricultural Research Organization, The Volcani Center, 68 HaMaccabim Road, P.O.B 15159, Rishon LeZion 7505101, Israel; chenklap@gmail.com (C.K.); neta.luria.8@gmail.com (N.L.); elishevasmith@gmail.com (E.S.); lior.hadad@mail.huji.ac.il (L.H.); elenab@agri.gov.il (E.B.); noa@volcani.agri.gov.il (N.S.); odedl@volcani.agri.gov.il (O.L.); diana@volcani.agri.gov.il (D.L.); 2 The Robert H. Smith Faculty of Agriculture, Food and Environment, The Hebrew University of Jerusalem, Rehovot 761001, Israel; 3 Department of Ornamental Plants and Agricultural Biotechnology, Agricultural Research Organization, The Volcani Center, 68 HaMaccabim Road, P.O.B 15159, Rishon LeZion 7505101, Israel; eddy@volcani.agri.gov.il

**Keywords:** synergism, in situ immunofluorescence, mixed infections

## Abstract

The tobamovirus tomato brown rugose fruit virus (ToBRFV), a major threat to tomato production worldwide, has recently been documented in mixed infections with the potexvirus pepino mosaic virus (PepMV) CH2 strain in traded tomatoes in Israel. A study of greenhouse tomato plants in Israel revealed severe new viral disease symptoms including open unripe fruits and yellow patched leaves. PepMV was only detected in mixed infections with ToBRFV in all 104 tested sites, using serological and molecular analyses. Six PepMV isolates were identified, all had predicted amino acids characteristic of CH2 mild strains excluding an isoleucine at amino acid position 995 of the replicase. High-throughput sequencing of viral RNA extracted from four selected symptomatic plants showed solely the ToBRFV and PepMV, with total aligned read ratios of 40.61% and 11.73%, respectively, indicating prevalence of the viruses. Analyses of interactions between the co-infecting viruses by sequential and mixed viral inoculations of tomato plants, at various temperatures, showed a prominent increase in PepMV titers in ToBRFV pre-inoculated plants and in mixed-infected plants at 18–25 °C, compared to PepMV-single inoculations, as analyzed by Western blot and quantitative RT-PCR tests. These results suggest that Israeli mild PepMV isolate infections, preceded by ToBRFV, could induce symptoms characteristic of PepMV aggressive strains.

## 1. Introduction

The tobamovirus tomato brown rugose fruit virus (ToBRFV) and the potexvirus pepino mosaic virus (PepMV) have been established as major disease-causing agents in tomatoes (*Lycopersicun esculentum*) worldwide. Recently, ToBRFV, identified in Jordan and Israel [1,2], has spread in the Middle East and to Europe, America and China [3,4,5,6,7,8,9,10]. Unlike ToBRFV, PepMV worldwide spread has long been established [11,12,13,14,15,16,17]. However, in Israel, we have only recently identified PepMV in symptomatic traded tomatoes, co-infected with ToBRFV [18].

Both ToBRFV and PepMV are single-stranded positive-sense RNA viruses (+ssRNA). ToBRFV particles have a rod like shape and are ~300 nm in length. The virus has a ~6.2 kb genome containing the four open reading frames (ORFs) encoding two subunits of RNA-dependent RNA polymerase (RdRp) complex: a short 126 kDa protein and a large 180 kDa protein; a movement protein (MP) and a ~17 kDa coat protein (CP) [2]. PepMV particles are filamentous and are 508 nm in length. The PepMV ~6.4 kb genome has five ORFs encoding the RdRp complex, triple gene block proteins and a ~26 kDa CP. There are four known PepMV genotypes: the Peruvian (LP), identified in pepino (*Solanum muricatum*), the European (EU), first identified in tomatoes, the Chilean (CH2), which commonly occurs in Europe, and the American (US1) [12,19,20,21,22,23,24].

Characteristic ToBRFV-induced symptoms are leaf narrowing and fruit bleaching [2], while PepMV infection induces a wide range of symptoms including leaf narrowing/bubbling, fruit marbling and the appearance of open unripe fruits [25,26]. PepMV symptom severity characteristics do not correlate with virus genotypes although enhanced symptom severity occurred upon mixed infections with PepMV mild and aggressive strains that belonged to different genotypes [27]. Environmental conditions, tomato cultivars and an enhanced virus accumulation are factors affecting symptom severity [28]. In addition, nonsynonymous nucleotide sequence variations have been shown to differentiate between aggressive and mild/attenuated PepMV strains [24,29,30,31,32,33]. It has also been shown that a single amino acid mutation in a mild strain could confer induction of a necrotic phenotype [30].

The PepMV recently identified in Israel in symptomatic tomatoes has been detected only in mixed infections with ToBRFV. Tomato plants co-infected by ToBRFV and PepMV have been reported [4,8]. However, a wide range of new distinctly severe symptoms, characteristic of PepMV aggressive strain single infections [24,25], was observed in greenhouse grown tomato plants co-infected by PepMV and ToBRFV. We have therefore isolated and sequenced the whole genome of the PepMV genotypes/strains occurring in symptomatic tomato plants in Israel. We have investigated whether an aggressive strain caused the severe disease symptoms or either co-occurrence of different PepMV genotypes or the co-infecting ToBRFV conferred enhanced symptom severity that could be associated with a high viral load in the symptomatic plants.

## 2. Materials and Methods

### 2.1. A Study of New Symptomatic Tomato Plants in Israel and ToBRFV and/or PepMV Inoculation Experiments

A study of field-collected symptomatic tomato plants was conducted in Israel in 2019 covering ~80% of the tomato-growing area. Three to five plants from each of the 104 plots were tested by enzyme-linked immunosorbent assay (ELISA) and sampled plants positive for ToBRFV and/or PepMV were further analyzed by RT-PCR followed by Sanger sequencing (see below). PepMV ELISA-positive tomato plant samples from six different disease phenotypes were also subjected to whole-genome sequencing (see below).

Inoculation studies were carried out with ToBRFV-IL [2] and/or PepMV-Ya isolate (MT018447), separated from ToBRFV by inoculating *Datura stramonium* plants, which showed necrotic local lesions in response to ToBRFV infections [2] and were systemically infected by PepMV [34]. Laboratory inoculation experiments were carried out under two different environmental temperature conditions: uncontrolled and controlled. Infectivity potential (see below) and an initial characterization of PepMV-Ya phenotypic effects were carried out under uncontrolled temperature conditions, with a temperature range of 25–45 °C. The viral effects on tomato leaf symptom development were studied by inoculating three different tomato plant cultivars—Zohara, Shiran (cherry) and Ikram—with ToBRFV, PepMV or a mixture of the two viruses, infecting five plants from each cultivar per inoculum. Inoculations were performed at the four to five leaf stage of the plants. Ratios of symptomatic leaves, showing various manifestations of the disease, per total leaf number in each inoculated plant, were calculated at 76 days post-inoculation (dpi).

Viral expression analyses in tomato plants inoculated by each virus separately or by a mixture of the viruses, were carried out under both uncontrolled temperatures (at 25 to 45 °C; two experiments), and controlled temperatures of 20 and 30 °C (three experiments). The two different inoculation experiments, conducted by infecting 2–3 tomato plants *cv*. Ikram per inoculum at the four to five leaf stage of the plants, were subjected to Western blot analyses for viral CP detection at 45 and 42 dpi, respectively (see below).

Analyses of viral gene expressions, using both Western blot and quantitative RT-PCR tests, and symptom development as well as synergy factor calculations of symptom severity (see below) were performed under three different controlled temperatures of 18, 25 and 30 °C. Tomato plants *cv*. Ikram were inoculated with either ToBRFV or PepMV for detection of single infection effects. For mixed-infection data, the plants were inoculated with a mixture of the viruses or sequentially, either by pre-inoculations with ToBRFV or pre-inoculations with PepMV. There was a period of one week between the sequential inoculations of the viruses. Four plants were inoculated with each viral preparation for each of the tested temperatures. The inoculated plants were tested at 58 dpi. For all analyses, the inoculations were performed at the four to five leaf stage of the plants and the third leaf below the apical meristem was sampled.

### 2.2. Infectious Potential

Tomato plants *cv.* Ikram, inoculated with ToBRFV, PepMV or a mixture of the viruses, served for infectivity potential analyses by measuring the extent of viral spread initiated by contaminated hands. The experiments (three experiments for each inoculum source) were carried out under uncontrolled temperatures, ranging between 25 and 45 °C. For hand contaminations, leaves from the various infected plants served as inoculum sources (confirmed by ELISA) and were crushed between fingertips. Viral transmission by contaminated hands was carried out by squeezing up to thirty new tomato plants consecutively. The percentage of infected plants was determined by inspecting symptom development and subjecting all newly touched plants to ELISA test at 15–21 dpi for detection of ToBRFV and/or PepMV.

### 2.3. PepMV Virion Purification and Specific Antiserum Preparation

In a recent documentation of PepMV in Israel, we have found the virus in mixed infections with ToBRFV [18]. In order to purify PepMV virions for specific antibody preparations and for single-tomato-plant inoculation studies, *D. stramonium* plants were inoculated with the ToBRFV and PepMV mixture source. While ToBRFV inoculations caused necrotic local lesions in this test plant [2], PepMV caused a systemic infection of the plant [34]. Systemically infected *D. stramonium* plant leaves (100 g) were ground in 100 mL 0.1 M potassium phosphate buffer, pH = 7.0, containing 0.5% sodium sulfite. A mixture of chloroform-butanol (1:1 *v/v*), comprising 10% of the leaf suspension, was added followed by incubation at 4 °C for an hour with stirring. The suspension was then centrifuged for 20 min at 13,000× *g* and the supernatant, filtered through Miracloth (Calbiochem-Behring Corp., La-Jolla, CA, USA), was ultra-centrifuged for 2.5 h at 200,000× *g*. The pellet, suspended in 1 mL 0.01 M potassium phosphate buffer pH = 7.0, was then placed on 4 mL sucrose 30% in 0.01 M potassium phosphate buffer pH = 7.0 and subjected to ultra-centrifugation for 2.5 h at 200,000× *g*. The pellet was suspended in 1 mL 0.01 M potassium phosphate buffer pH = 7.0 and a sample was analyzed by Western blot for the presence of PepMV only, using specific antibodies for PepMV (AS-1022, kindly provided by Wulf Menzel, Leibniz Institute DSMZ-GmbH, Braunschweig, Germany) and ToBRFV [2]. Transmission electron microscopy analysis, performed, as previously described [18], confirmed the presence of potexvirus like particles in the virion preparation and particles characteristic of the Tobamovirus genus were not observed. The purified PepMV virions served for antiserum preparation, as previously described [2], performed by Adar Biotech LDT, Rehovot, Israel (Order No. 4501746647, April 29th, 2019).

### 2.4. Indirect Enzyme-Linked Immunosorbent Assay (ELISA)

Symptomatic tomato leaves and fruits as well as ToBRFV- and/or PepMV-inoculated tomato plant leaves were analyzed by indirect ELISA test, as previously described [18]. Antiserum specific for ToBRFV [2] or PepMV (current study), using 1:5000 dilutions in PBS, served for viral detection. Optical density (O.D.) values with a minimum ratio of 3-fold the negative control were considered positive.

### 2.5. Viral RNA Extraction, Reverse Transcription (RT) and PCR Amplification

Viral RNA extraction was performed using Accuprep Viral RNA Extraction kit (Bioneer, Daejeon, Korea). The qPCRBIO cDNA synthesis kit (PCR Biosystems, London, UK) was employed and the obtained cDNA was subjected to PCR amplification. The primer sets used for identification of ToBRFV or PepMV were 1 and 2 (Table 1), respectively. The primer sets used for PepMV whole-genome sequencing were 3–9 (Table 1). Obtained amplicons were sequenced using Sanger sequencing (HyLabs, Rehovot, Israel) and sequence confirmation of genome segments or whole genome was performed using the search algorithm Basic Local Alignment Search Tool (BLAST) against the National Center for Biotechnology Information (NCBI) GenBank (https://blast.ncbi.nlm.nih.gov/Blast.cgi).

### 2.6. High-Throughput Sequencing (HTS) with Illumina HiSeq Platform and Bioinformatic Analysis

Field-collected symptomatic tomato fruits and leaves from four plants, showing one distinctive phenotype, were combined and subjected to viral RNA extraction using Accuprep Viral RNA Extraction kit (Bioneer, Daejeon, Korea). The RNA extract served for library construction with ScriptSeq Complete kit (Plant Leaf, Illumina, San Diego, CA, USA). Library sequencing was performed by the use of Illumina Hiseq 2500 (50 cycles) (Technion Genome Center, Haifa, Israel). Quality filtering of the raw RNAseq reads was performed with Trimmomatic version 0.32 software. VirusDetect software version 1.7 was employed for clean read search, using the plant virus database with the software pipeline default parameters [35]. For genome assembly, de novo assembly was combined with mapping to plant virus sequences from GenBank, using velvet [36] and bwa [37] for read mapping. Calculations of depth and coverage of the virus genome were performed using bowtie2 [38] and SAMtools [39] with the default SAMtools software parameters. The reference viral genome for the calculations was the fully assembled genome validated by RT-PCR. The percent nucleotide sequence identity was calculated using the GenBank BLASTn search tool. The deduced amino acid (aa) sequence alignment of the six PepMV isolates was performed using Multalin sequence alignment software [40]. Pairwise deduced aa sequence alignment and identity calculations were performed using SDTv1.2 software [41].

### 2.7. Western Blot Analyses

Tomato leaves from ToBRFV- and/or PepMV-inoculated plants were subjected to protein extractions and Western blot analyses, as previously described [18]. Protein extractions were performed using USB extraction buffer (75 mM Tris-HCL (pH 6.8), 8 M urea, 4.5% (*g/v*) SDS and 7.5% (*v/v*) ß-mercaptoethanol) while keeping a constant ratio of 1 μg/5.5 μL between leaves and buffer. Proteins were separated on 15% SDS-PAGE and the blotted nitrocellulose membranes were subjected to viral CP detection using specific polyclonal antibodies for ToBRFV [2] and PepMV (current study) with 1:4000 dilution ratios in phosphate-buffered saline (PBS). Ponceau-S staining of total loaded proteins was performed following the CP detection with alkaline-phosphatase conjugated goat anti-rabbit antibodies in order to prevent colored background in the CP data. ImageJ software was used to estimate CP specific band intensities. Statistical significance of differences between CP band intensities were calculated using *t*-Test: Two sample assuming unequal variances.

### 2.8. In Situ Immunofluorescence

In situ immunofluorescence was carried out, as previously described [18]. Leaves from tomato plants *cv*. Ikram with mixed infections of ToBRFV and PepMV were subjected to analysis. Specific antisera for ToBRFV [2] and PepMV (current study) were used. For detection of both viruses in leaves of mixed-inoculated plants, a high concentration of unlabeled goat anti-rabbit antibodies (SIGMA, A9919, 1:100 dilution in PBS containing 2% milk) was added after binding of the first antibody (anti-ToBRFV) and its specific anti-rabbit IgG conjugated to Alexa Fluor 488, and before adding anti-PepMV antibodies, in order to block all unbound ToBRFV antibodies. The appropriate concentrations of unlabeled goat anti-rabbit antibodies were tested separately and confirmed as good for this blocking purpose. The samples were then washed with PBS solution containing 0.05% (*v/v*) Tween-20 (PBS-T) before proceeding to the PepMV-specific binding analysis. The labeled secondary antibodies were goat anti-rabbit IgG conjugated either to Alexa Fluor 488 for ToBRFV detection or to Alexa Fluor 594 for PepMV detection (Invitrogen, Carlsbad, CA, USA).

### 2.9. Quantitative RT-PCR (RT-qPCR)

Leaves from ToBRFV-, PepMV- or mixed-infected tomato plants (50–100 mg) were subjected to total RNA extraction using a TRI Reagent kit (MRC, Inc., Cincinnati, OH, USA). RNA concentrations were measured by a spectrophotometer NanoDrop ND1000 (Thermo Scientific, Wilmington, DE, USA). cDNA synthesis was performed on 1 µg of total RNA using a Verso™ cDNA Kit (Thermo Fisher Scientific, Epsom, UK) with the oligo (dT) primer (10 pmol/µL). RT-qPCR was performed using the power SYBR Green PCR master MIX (Applied Biosystems, Thermo Fisher scientific, Vilnius, Lithuania) and running was performed using the StepOnePlus^TM^ (Applied Biosystems, Fisher Scientific Company, Ottawa, Ontario). The tomato endogenous gene *TIP41* served as a reference gene [42] and was analyzed with each tested batch of viruses. Primers for the reference gene *TIP41* and the two target genes—ToBRFV-CP and PepMV-CP—were designed with Primer3 Plus software. The primer set for ToBRFV-CP was F 5′ CACAATCGCAACTCCATCGC 3′ and R 5′ CAGGTGCAGAGGACCATTGT 3′, amplicon size of 159 bp; for PepMV-CP was F 5′ GTGCACTTGCTGCACAGTTT 3′ and R 5′ GGTGGCACATTGCTGTCTAA 3′, amplicon size of 106 bp and for *TIP41* was F 5′ ATGGAGTTTTTGAGTCTTCTGC 3′ and R 5′ GCTGCGTTTCTGGCTTAGG 3′, amplicon size of 235 bp. The amplification of the tested viruses was performed in duplicates with the specific primers. Each sample was analyzed against the *TIP41* endogenous gene. Each reaction contained 100 ng cDNA (cDNA reverse transcribed from 100 ng RNA) in a 15 µL reaction mixture containing 4 µL of diluted cDNA, 3 pmols of each primer and 7.5 µL Absolute QPCR Sybr Green Mix (Thermo Fisher Scientific, Vilnius, Lithuania ). Reaction conditions were: 10 min at 95 °C (“hot start”) followed by 40 cycles of 3 sec at 94 °C, 15 sec at 60 °C, and 20 sec at 72 °C. The quantitative analysis was performed using the StepOnePlus^TM^ bio system (Applied Biosystems, Fisher Scientific Company, Ottawa, Ontario). The percent amplification efficiency of each of the analyzed samples equaled: 1%. ΔCt, obtained by subtracting Ct of the endogenous gene from Ct of the tested virus, was calculated for each tested virus in all analyzed samples. ΔΔCt was calculated by subtracting mean ΔCt of each virus in the single infection samples from each ΔCt of the respective mixed-infection samples. The difference between viral ΔCt of the various mixed infections and that of single infections (comprising the derived specific ΔΔCt), at each of the three tested temperatures, was subjected to *t*-Test: two-sample assuming unequal variances. ΔΔCt of each mixed-infection sample served for calculation of 2−ΔΔCt for estimation of relative gene expression in the mixed-infection samples relative to the respective single infection samples [43,44]. The mean 2−ΔΔCt ± the standard deviation of the mean (s.d.) data for each gene in the various tested samples were graphed.

### 2.10. Synergy Factor (SF) Calculations

ToBRFV and PepMV co-infection effects on the proportional change in tomato plant growth or viral load, measured by Western blot analysis, were subjected to synergy factor (SF) calculations by applying the Abbott method for an independent joint action assumption [45,46]. The synergistic interaction between the viruses was indicated by a higher value of the observed disease effects of the co-infecting viruses (Eobs) when compared to the expected effects calculated from the disease symptoms caused by each virus separately (Eexp). Eexp is calculated according to the equation:

Eexp (%) = A + B − (AB/100)(1)

where A and B are the plant proportional response to a single infection by ToBRFV or PepMV, respectively. The synergistic interaction is calculated according to the equation:

SF = Eobs/Eexp(2)

where Eobs is the mean of the results obtained under the mixed-infection procedures. It is important to note however, that at high response levels to co-infections, this calculation method would show low synergy factor values [47]. For calculations of phenotypic synergy between the viruses, mean percent reduction in plant heights or number of leaves by each virus separately, were A and B in Equation (1) for each phenotype, and Eobs for Equation (2) was the mean observed change in those parameters in mixed-infected plants. For calculations of synergy in total viral load, mean proportional viral load values were estimated by measurements of the viral CP band intensity for each virus, calculated from Western blot data using ImageJ software, as a percentage of a constant plant protein band, observed in the same Western blot membrane subjected to Ponceau-S staining of total loaded proteins. The mean proportional viral CP values obtained in ToBRFV or PepMV single inoculations were A and B in Equation (1), respectively, and the mean proportional total viral load (CP of both viruses combined) in the mixed-infected plants was Eobs in Equation (2).

## 3. Results

### 3.1. ToBRFV and PepMV Co-Infections in Distinctly Severe Symptomatic Tomato Plants

**Figure 1:**
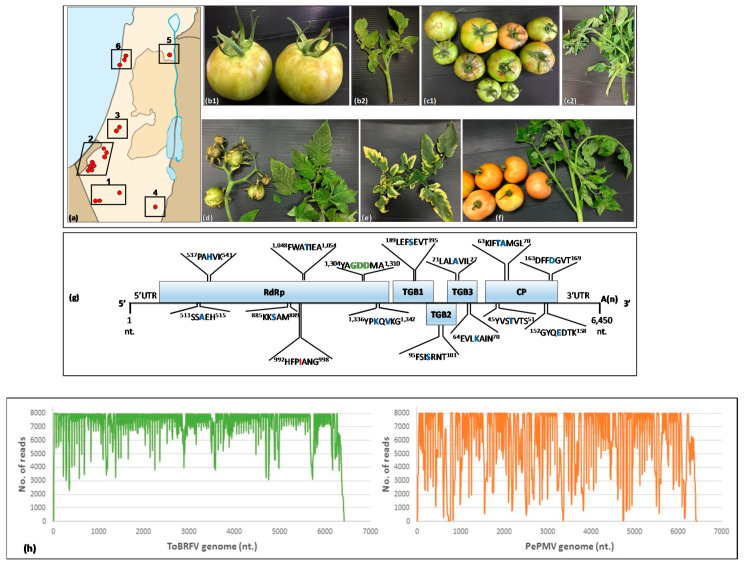
New severe symptomatic tomato plants observed in tomato-growing plots widely spread in Israel showed mixed infections with pepino mosaic virus (PepMV) and tomato brown rugose fruit virus (ToBRFV-IL). (**a**) A geographical scheme of the tomato-growing sites in Israel affected by the new disease symptoms: 1, Ramat Negev; 2, Bsor; 3, Lakhish; 4, Arava; 5, Beit She’an Valley; 6, Hefer Valley. (**b**)–(**f**) Symptomatic tomato plants. (**b1**) Brown rugose spots on unripe fruits. (**b2**) Mild mottling on leaves. (**c1**) Scarred and open unripe fruits. (**c2**) Leaf narrowing. (**d**) Open undeveloped fruits and interveinal chlorosis on leaves. (**e**) Bright yellow patched leaf margins. (**f**) Bright yellow patched and marbled fruits and mosaic on fern leaves. (**g**) A scheme of PepMV genome depicting open reading frames and selected deduced amino acids (aa) shared by all the Israeli isolates (GenBank accession Nos.: MT018444, MT018445, MT018446, MT018447, MT018448, MT018449). Blue-colored aa are characteristic of mild PepMV CH2 strains; a red-colored aa is characteristic of an aggressive PepMV CH2 strain; a green-colored aa triplet is a conserved sequence shared by both aggressive and mild PepMV CH2 strains; RdRp, RNA-dependent RNA polymerase; CP, coat protein. (**h**) High-throughput sequencing of PepMV-Ah isolate and ToBRFV-IL isolate (GenBank accession Nos. MT018444 and KX619418, respectively) found in symptomatic tomato plants collected from Ahituv (Hefer Valley). Read Nos. and depths covering the whole genome of either PepMV or ToBRFV are depicted.

In recent years, ToBRFV has spread widely in the tomato-growing locations in Israel, displaying disease symptoms in 10–30% of the harvested fruits. Recently, distinctly severe viral disease symptoms have occurred in greenhouse-grown tomato plants in Israel, resulting in scarred or open unripe fruits along with narrow or yellow patched leaves (Figure 1).

A study of greenhouse tomato plants conducted in Israel in 2019, covering ~80% of the tomato-growing area, revealed a wide occurrence of the new symptom type in tomato plants. These new symptoms featured characteristics of disease caused by PepMV aggressive strain infection of tomatoes [24,25]. Three to five plants from each of the 104 tomato-growing plots were sampled and analyzed first by ELISA, and positive samples were confirmed by RT-PCR followed by Sanger sequencing. The results revealed the presence of both ToBRFV and PepMV in the severe symptomatic tomato plants (Table 2). The ELISA-obtained O.D. value range for the infected plants was 0.6–2.8 for ToBRFV and 1.3–3.5 for PepMV, while the control value range was 0.002–0.025. Tomato plants co-infected by ToBRFV and PepMV have been reported [4,8] and we have recently documented the new detection of PepMV in Israel [18]. Considering the wide spread incidence of ToBRFV-infected tomato plants in Israel, the co-occurrence of ToBRFV and PepMV in the symptomatic plants was not surprising. However, the wide spread of the extremely severe symptoms led us to ask whether an aggressive PepMV strain, co-infection of various PepMV strains or ToBRFV in the mixed-infected plants was the causal factor for the severe disease.

### 3.2. Genome Characterization of PepMV Isolates in Symptomatic Tomato Plants Co-Infected by ToBRFV and PepMV

Field-collected co-infected tomato plants, demonstrating six distinctly different severe phenotypes, were subjected to PepMV whole-genome sequencing using Sanger sequencing of RT-PCR amplicons along the genomes (five phenotypes) and HTS (one phenotype). Each phenotype was studied by combining fruits and leaves from three to four symptomatic tomato plants, which showed the distinctive phenotype and were ELISA and RT-PCR positive for both ToBRFV and PepMV. Six different PepMV isolates were identified (GenBank accession Nos.: MT018444, MT018445, MT018446, MT018447, MT018448, MT018449). Importantly, HTS of the viral RNA preparation showed prevalence of ToBRFV and PepMV in the tested symptomatic plants (Figure 1h). Total read number was 33,512,845. Reads aligned exactly one time on the ToBRFV genome were 13,610,178, comprising 40.61% overall distribution alignment rate. Reads aligned exactly one time on the PepMV genome were 3,931,318, comprising 11.73% overall distribution alignment rate. No other plant viruses were detected. The six PepMV isolates were highly similar, showing 99.25–99.58% nucleotide sequence identity, covering 99% of the genome. All newly identified PepMV isolates showed 98.32–98.58% nucleotide sequence identity with PepMV CH2 strains, with 99% genome coverage. Deduced aa sequence of the six PepMV Israeli isolates were aligned showing aa sequence identity of 99.2–99.8% in the RdRp, 98.7–100% in the CP, 98.7–100% in TGB1, 100% in TGB2 and 98.8–100% in TGB3, with the differential aa listed in Table S1. Importantly, analyses of the deduced aa for the presence of aa characteristic of aggressive strains [24,29,30,31,32,33] showed that the six Israeli isolates were identical. We have found that excluding an isoleucine at the ^992^ HFPIANG ^998^ domain, at the aa position 995 of the replicase coding region, previously found in an aggressive PepMV CH2 strain [25], all other reported aa characteristic of attenuated or mild PepMV strains were encoded by all the Israeli isolates (Figure 1g). In addition, the conserved GDD triplet at the polymerase domain of the replicase, associated with a conditional necrotic phenotype induction [28], was encoded by all PepMV isolates as well (at the deduced aa position 1306–1308). Importantly, in light of a previous report that a single aa mutation in a mild PepMV CH2 strain could confer aggressive characteristics to the mild strain [30], it was imperative to determine whether the Israeli PepMV isolates could be classified as mild strains.

### 3.3. Biological Assays and Serological Tests Showing PepMV Mild Effects in Singly Infected Tomato Plants Turn Aggressive in Mixed Infections with ToBRFV

**Figure 2:**
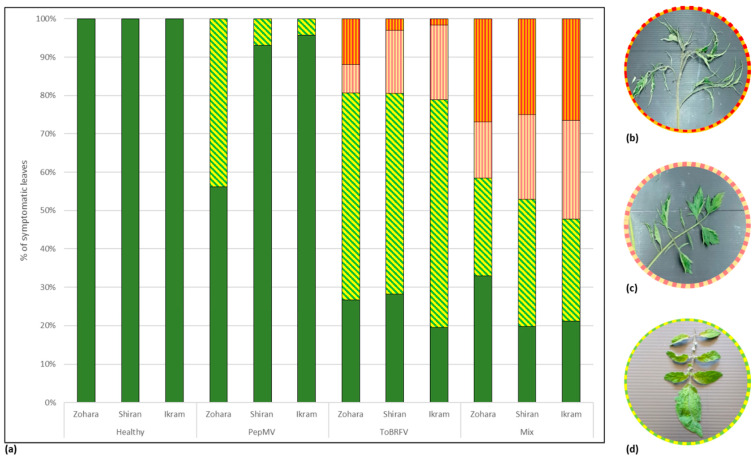
Enhanced severity of symptom development in tomato brown rugose fruit virus (ToBRFV) and pepino mosaic virus (PepMV) mixed-inoculated tomato plants compared to singly inoculated plants. (**a**) A graphical depiction of mean percent symptomatic leaves in each of three tomato plant cultivars: Zohara, Shiran and Ikram either singly inoculated with ToBRFV or PepMV, or co-inoculated with a mixture of the viruses. The various leaf manifestations are color coded. A green color indicates healthy leaves; a yellow color with green slanted stripes indicates mosaic yellowing leaves; a bright orange color with vertical stripes indicates mosaic serrated leaves; a dark orange color with vertical stripes indicates a shoestring-like phenotype. (**b**) Shoestring-like leaves. (**c**) Mosaic serrated leaves. (**d**) Mosaic yellowing leaves.

Characterization of the new PepMV isolate was carried out by inoculating three different cultivars of tomato plants: Zohara, Shiran (cherry) and Ikram, with PepMV-Ya isolate (MT018447), either alone or in a mixture with ToBRFV-IL. PepMV-Ya isolate was extracted from systemically infected *D. stramonium* plants. The inoculated tomato plants were grown in a greenhouse under uncontrolled temperature conditions (at a temperature range of 25–45 °C). The ELISA O.D. value range of the infected plants was 0.3–3.1 for ToBRFV, 0.2–3.4 for PepMV while the O.D. value range of the control was 0.04–0.08. Leaf symptom manifestations were rated as degrees of severity, the highest indicated the appearance of shoestring-like leaves; milder manifestations were leaves with serrated margins and the lowest severity indicated the appearance of mosaic or bright yellowing leaves (Figure 2). The percentages of the various symptomatic leaves per plant in each cultivar under three viral inoculation conditions were graphed. The results clearly showed that in all three tested cultivars, disease symptoms induced by PepMV single inoculations were of the lowest severity, showing only mosaic/bright yellowing leaves, which amounted to 44 ± 33% s.d. (*n* = 30), 7 ± 8% s.d. (*n* = 30) and 4 ± 10% s.d. (*n* = 33), of the total leaves in Zohara, Shiran and Ikram, respectively. Interestingly, the ratios of mild manifestations of serrated leaves, amounting to 7 ± 6% s.d. (*n* = 96), 16 ± 6% s.d. (*n* = 125) and 19 ± 3% s.d. (*n* = 134) of the total leaves in ToBRFV singly inoculated Zohara, Shiran and Ikram plants, respectively, were 1.4- to 2.1-fold higher in ToBRFV and PepMV mixed-infected tomato varieties. Importantly, very low ratios of symptomatic leaves showing the most severe shoestring-like phenotype were observed in ToBRFV singly inoculated plants but the ratios were high in ToBRFV and PepMV co-inoculated plants. ToBRFV singly inoculated Zohara, Shiran and Ikram plants showed shoestring-like leaf ratios of 12 ± 9% s.d., 3 ± 5% s.d. and 2 ± 2% s.d, respectively, while the ToBRFV and PepMV co-inoculated cultivars showed high ratios of 27 ± 7% s.d. (*n* = 113), 25 ± 9% s.d (*n* = 134) and 26 ± 10% s.d. (*n* = 133), respectively. These results suggest that regarding leaf symptom development, the Israeli PepMV isolate is a mild strain, and these inoculation studies seemed to recapitulate the severe effects of ToBRFV and PepMV mixed infections.

**Figure 3:**
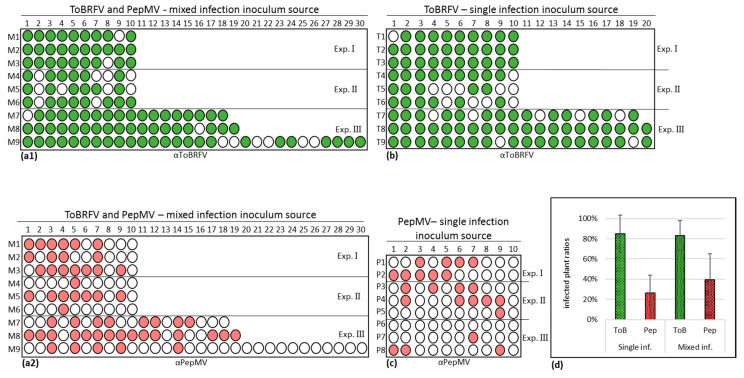
Infectious potential of tomato brown rugose fruit virus (ToBRFV) and pepino mosaic virus (PepMV) singly and mixed-infected tomato plants. (**a**)–(**d**) Depiction of enzyme-linked immunosorbent assay results. (**a1**) A spread range of ToBRFV from ToBRFV and PepMV mixed-infection inoculum source (*n* = 127). (**a2**) A spread range of PepMV from ToBRFV and PepMV mixed-infection inoculum source (*n* = 127). (**b**) A spread range of ToBRFV from ToBRFV single infection inoculum source (*n* = 119). (**c**) A spread range of PepMV from PepMV single infection inoculum source (*n* = 80). (**d**) A graphical depiction of calculated mean percentages of infected plants. Bars represent the standard deviation of the mean. Exp., experiment; ToBRFV detection results are green colored; PepMV detection results are pin colored; ToB, ToBRFV; Pep, PepMV; inf, infection; *n*, number of total plants.

In order to confirm that the PepMV singly inoculated tomato plants harbor viable viruses and to analyze the possible contribution of ToBRFV and PepMV mixed infections to each virus spread range, we tested the infectious potential of the various inoculated tomato plants and measured the extent of viral spread. Contaminated hand transmission was carried out by crushing infected leaves (ELISA positive) of the three-inoculum sources between fingertips followed by squeezing up to thirty new plants, consecutively in order to measure viral spread range. All the touched tomato plants in the various experiments were tested by ELISA, showing an O.D. value range of 0.128–2.85 for ToBRFV and 0.093–2.065 for PepMV compared to 0.0009–0.031 of the control. The data presented in Figure 3 showed that PepMV from the PepMV singly infected tomato plant inoculum source had a low spread range, amounting to 26.25 ± 18% s.d. of the plants (*n* = 80). The range of tomato plant infection by ToBRFV from singly and ToBRFV and PepMV mixed-infected plants was high, showing infection ratios of 84.85 ± 18% s.d. (*n* = 119) and 83.22 ± 15% s.d. (*n* = 127), respectively. The infection ratio of PepMV from ToBRFV and PepMV mixed-infected plants was 1.5-fold higher (the factor refers to mean ratios, *n* = 127) than that from PepMV singly infected plant inoculum source. Importantly, monitoring leaf symptom development showed that during the fourteen days of the conducted experiments PepMV singly infected plants did not show any viral symptoms. Unlike PepMV, ToBRFV singly infected plants as well as ToBRFV and PepMV mixed-infected plants were symptomatic. ToBRFV singly infected plants showed severe mottling and serrated leaves while mixed-infected plants (ToBRFV and PepMV co-infected) showed additional fern leaf symptoms.

**Figure 4:**
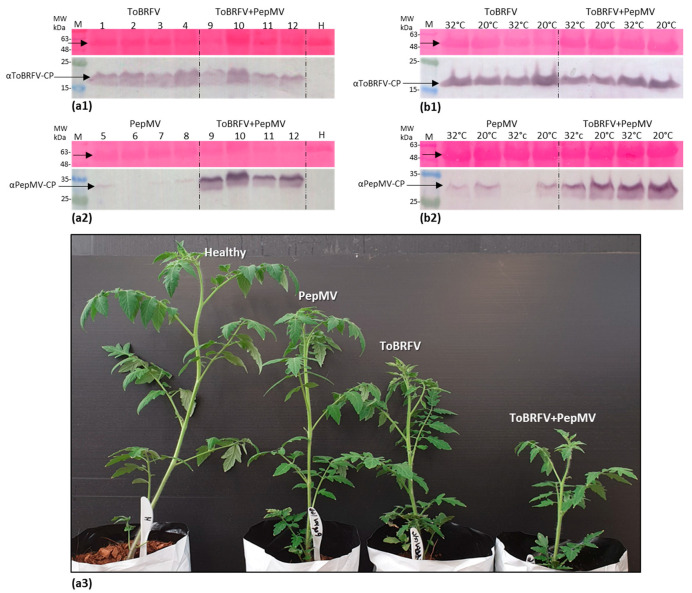
Enhanced pepino mosaic virus (PepMV) titers in tomato brown rugose fruit virus (ToBRFV) and PepMV co-inoculated tomato plants compared to PepMV singly inoculated plants. (**a1**,**2**) Western blot analyses showing coat protein (CP) levels of ToBRFV and PepMV in singly and mixed-inoculated tomato plants grown under uncontrolled temperatures (at a temperature range of 25–45 °C). (**a3**) A prominent reduction in plant heights observed in the mixed-inoculated tomato plants grown at a temperature range of 25–45 °C. (**b1**,**2**) Western blot analyses showing CP levels of ToBRFV and PepMV in singly and mixed-inoculated tomato plants, grown under controlled temperatures of 20 and 32 °C. H, healthy controls; M, molecular size marker; arrows indicate the two viral CPs and a constantly expressed plant protein stained by Ponceau-S dye.

The low infectious potential of PepMV singly infected tomato plants could reflect a low virus titer. Therefore, the viral CP expression levels were analyzed by Western blot of the various viral inoculated plants grown under uncontrolled temperature conditions (at 25–45 °C). The obtained results revealed that PepMV CP levels in PepMV singly inoculated plants were very low when compared to PepMV CP levels in the ToBRFV and PepMV mixed-inoculated plants, which were 6.55-fold higher (*n* = 4) (Figure 4a).

**Figure 5:**
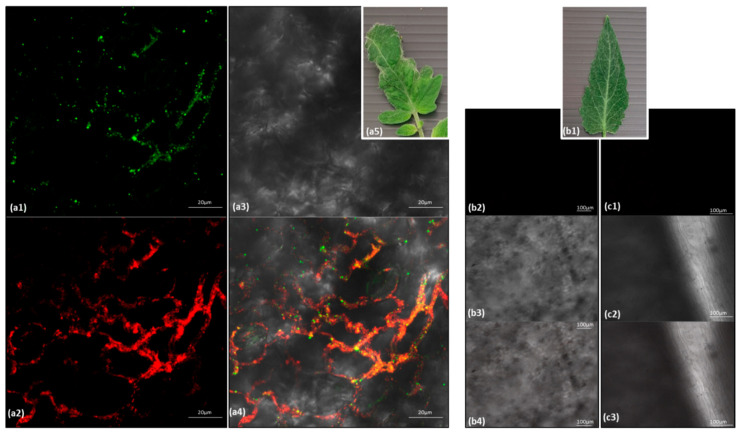
In situ immunofluorescence imaging of tomato brown rugose fruit virus (ToBRFV) and pepino mosaic virus (PepMV) in mixed-inoculated tomato plants. (**a1**) ToBRFV coat protein (CP) detection in mixed-inoculated tomato plant leaves, using antibodies conjugated to Alexa 488, visualized with a green channel. (**a2**) PepMV CP detection in mixed-inoculated tomato plant leaves, using antibodies conjugated to Alexa 594, visualized with a red channel. (**a3**) Mixed-inoculated tomato plant leaves visualized using a bright field. (**a4**) ToBRFV and PepMV detection in mixed-inoculated tomato plant leaves, using merged channels. (**a5**) A symptomatic leaf of mixed-inoculated tomato plants, showing mosaic and leaf narrowing and a deformity. (**b1**) A healthy tomato leaf. (**b2**) Visualization of a healthy tomato leaf using a green channel. (**b3**) Visualization of a healthy tomato leaf using a bright field. (**b4**) Visualization of a healthy tomato leaf using merged channels. (**c1**) Visualization of a healthy tomato leaf using a red channel. (**c2**) Visualization of a healthy tomato leaf using a bright field. (**c3**) Visualization of a healthy tomato leaf using merged channels.

ToBRFV CP levels remained similar in single and mixed-infected plants. Low PepMV CP levels could be indicative of impairment of replication efficiency and/or viral systemic spread. Therefore, PepMV responsiveness to temperature reduction was tested by conducting similar inoculation experiments under controlled temperature conditions (in three repeats). Western blot analyses showed that upon temperature reduction from 32 to 20 °C, PepMV CP levels in PepMV singly infected plants increased by a factor of 3.0 ± 1.7 s.d. (*n* = 4) (Figure 4b, showing a representative Western blot data). However, under these low temperature conditions (at 20 °C), there was still a prominent increase in PepMV CP levels in ToBRFV and PepMV mixed-inoculated plants when compared to PepMV single inoculations, amounting to a factor of 5.56 ± 4.42 s.d. (*n* = 14) (Figure 4b2). A study of ToBRFV and PepMV localization in leaves of the mixed-infected tomato plants showed co-localization of the two viruses (Figure 5).

### 3.4. PepMV Inoculations of Tomato Plants, Preceded by ToBRFV, Resulted in a High PepMV Titer Compared to PepMV Single Inoculations

High PepMV titers, presented by the viral CP detection, in the ToBRFV and PepMV mixed-inoculated tomato plants compared to PepMV singly inoculated plants, could be the causal factor of the severe disease symptoms observed in the field. To characterize the contribution of ToBRFV to manifestations of high PepMV titer levels and increased plant symptom severity in ToBRFV and PepMV mixed-infected tomato plants, viral inoculation experiments were conducted employing two different ToBRFV and PepMV sequential inoculation procedures, at three different temperatures. The rated tomato plant leaf-symptom severity observed under all experimental conditions were summarized in Table 3. 

**Figure 6:**
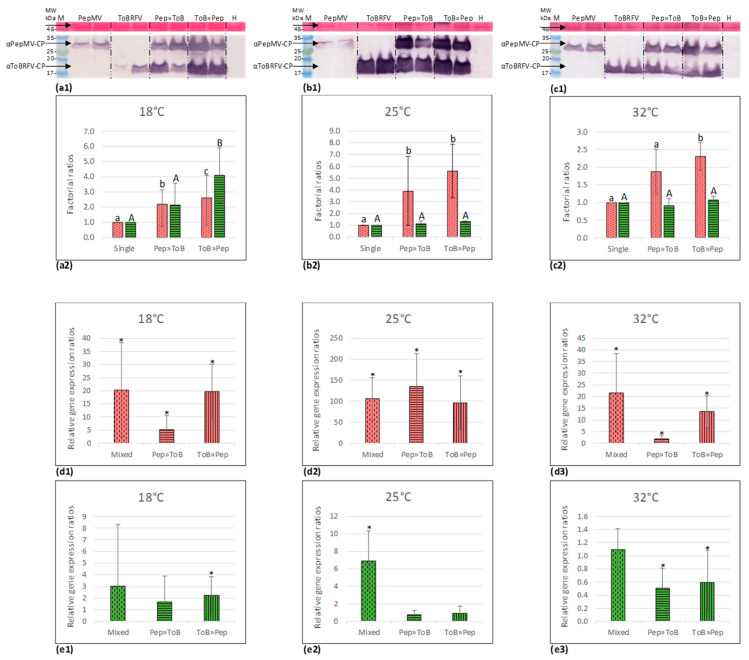
Enhanced pepino mosaic virus (PepMV) titers in tomato plants, successively inoculated with tomato brown rugose fruit virus (ToBRFV) and PepMV, compared to PepMV singly inoculated plants, occurred at both low and high environmental temperatures. (**a**)–(**c**) Western blot analyses results. (**d**), (**e**) Graphical depictions of viral relative gene expression ratios (2^-ΔΔCt^) calculated from quantitative RT-PCR results. (**a1**) A Western blot analysis showing coat protein (CP) levels of ToBRFV and PepMV in singly and various mixed-inoculated tomato plants grown at 18 °C. (**a2**) A graphical depiction of a calculated mean factorial increase in viral CP levels in the two sequentially mixed-inoculated plants compared to singly inoculated plants grown at 18 °C, using ImageJ software for band intensity measurements (*n* = 4). (**b1**) A Western blot analysis showing ToBRFV and PepMV CP levels in singly and various mixed-inoculated tomato plants grown at 25 °C. (**b2**) A graphical depiction of a calculated mean factorial increase in viral CP levels in the two sequentially mixed-inoculated plants compared to singly inoculated plants grown at 25 °C (*n* = 4). (**c1**) A Western blot analysis showing ToBRFV and PepMV CP levels in singly and various mixed-inoculated tomato plants grown at 32 °C (*n* = 4). (**c2**) A graphical depiction of a calculated mean factorial increase in viral CP levels in the two sequentially mixed-inoculated plants compared to singly inoculated plants grown at 32 °C (*n* = 4). H, healthy control; M, molecular size marker; arrows indicate the two viral CPs and a constantly expressed plant protein stained by Ponceau-S dye. Dotted columns represent PepMV results; striped columns represent ToBRFV results; bars indicate the standard deviation of the mean; a, b, c, indicate statistically significant PepMV results between the marked columns, *p* < 0.05; A, B, C, indicate statistically significant ToBRFV results between the marked columns, *p* < 0.05; statistical analyses: *t*-Test: Two sample assuming unequal variances. (**d1**–**3**) PepMV relative gene expression ratios in the various mixed-infected tomato plants at three different environmental temperatures. (**e1**–**3**) ToBRFV relative gene expression ratios in the various mixed-infected tomato plants at three different environmental temperatures. Asterisks (*) indicate a significant increase in viral relative gene expression ratios (*p* < 0.05), determined by subjecting the difference between viral ΔCt obtained in mixed infections and that obtained in single infections (comprising the derived viral ΔΔCt), to *t*-Test: Two sample assuming unequal variances. PepMV results are pink colored; ToBRFV results are green colored; ToB, ToBRFV; Pep, PepMV; *n*, number of total plants.

Western blot analyses showed that under all temperature conditions, PepMV CP levels were significantly higher in mixed-infected plants obtained by ToBRFV pre-inoculations, when compared to PepMV singly inoculated plants (Figure 6a–c). The factorial ratios were higher at 18 and 25 °C when compared to 32 °C. Interestingly, at 18 and 25 °C, the enhanced PepMV CP levels in the mixed-inoculated plants were significantly higher than the levels in PepMV singly inoculated plants irrespective of the inoculation order (Figure 6a2–c2). In addition, specifically at 18 °C, mixed-infected plants obtained by ToBRFV pre-inoculations showed significantly higher levels of ToBRFV CP when compared ToBRFV single inoculations (Figure 6a2). Analyses of relative gene expression ratios in the various mixed-infected plants using RT-qPCR revealed that at 18 °C the 2^−ΔΔCt^ values (calculated relative to the respective singly infected plants) reflected exactly the Western blot data at this temperature showing high PepMV expression levels in mixed-infected plants irrespective of viral inoculation order (Table S2, Figure 6d1). In addition, at 18 °C in ToBRFV pre-inoculated plants, significantly higher PepMV expression ratios were observed compared to PepMV pre-inoculated plants, concomitant to an increase in ToBRFV relative gene expression ratios (Figure 6d1,e1). Regarding viral inoculation order, both RT-qPCR and Western blot data showed that at the two extreme temperatures—18 and 32 °C—ToBRFV pre-inoculations showed a significantly higher effect on PepMV enhanced expression compared to the PepMV pre-inoculation procedure (Figure 6a2,c2,d1,d3). Particularly noteworthy is the positive correlation observed at 18 °C between the enhanced PepMV expression and high symptom severity in the ToBRFV pre-inoculated plants when compared to PepMV pre-inoculation (Table 3). This correlation did not occur in the ToBRFV pre-inoculated plants that were kept at 32 °C, which also showed low levels of ToBRFV (Table 3, Figure 6e3).

### 3.5. Synergy Factor (SF) for ToBRFV and PepMV Interactions

The significantly higher PepMV CP levels and relative gene expression ratios (2^−ΔΔCt^) as well as the increase in the severe shoestring-like leaf symptoms observed in the ToBRFV and PepMV mixed-infected tomato plants (Figure 2, Figure 6 and Table S2) were significant manifestations of synergy between the viruses. However, for calculations of SF, estimates of relative proportions in plant response under each inoculation condition were necessary [45,46]. Subjecting the proportional change in plant heights and leaf number to SF calculations showed that at 18 °C ToBRFV and PepMV mixed-infected plants, obtained by either inoculating the plants with a mixture of the viruses or by successive inoculations of PepMV and ToBRFV, had SF > 1 for both parameters (Table 4). We have also subjected the proportional viral load to SF calculations by estimating viral CP levels, obtained in Western blots, as a proportion of a constantly expressed plant protein, detected on the same blotted membrane by Ponceau-S total protein staining. Data observed in Figure 4a2 and Figure 6a were subjected to the calculations. The results clearly showed that in all tested ToBRFV and PepMV mixed-infected plants the combined proportional viral CP values (of both ToBRFV and PepMV) were higher than expected from the values obtained by each virus in the singly infected plants, showing SF > 1, indicating synergistic interactions between the viruses (Table 4).

## 4. Discussion

ToBRFV has been established in Israel as a major pathogen of tomato production, ever since 2014, showing characteristic symptoms familiar to growers. In 2019, new distinctly severe symptoms were reported in tomato plant-growing areas in Southern Israel. A study of greenhouse tomato plants was therefore conducted covering ~80% of the tomato-growing area in Israel. This study showed that severe symptoms were widespread in open or scarred unripe fruits with leaves showing various disease phenotypes including bubbling, yellow patches, narrowing or serrated margins. Interestingly, all the observed severe symptoms were associated with ToBRFV and PepMV co-infections. Importantly, none of the symptomatic tomato plants collected in the study were PepMV singly infected. Whole-genome sequence analyses of six different phenotypic manifestations of the new disease revealed the occurrence of six different PepMV isolates, which were highly similar showing ~99% nucleotide sequence identity and 98.7–100% deduced aa sequence identity. All PepMV new isolates showed ~98% aa sequence identity with CH2 strains. Although differing in up to 24 aa residues (Table S1), all six Israeli isolates had the characteristic aa of attenuated or mild PepMV strains, excluding the isoleucine aa at position 995 of the RdRp, characteristic of a CH2 aggressive strain (Figure 1) [25,30,33]. Most importantly, in laboratory analyses of phenotypic characteristics of a PepMV Israeli isolate, we have shown that three tomato plant cultivars inoculated with the PepMV-Ya isolate did not show any of the leaf symptoms characteristic of aggressive PepMV strains [25] suggesting that the Israeli PepMV isolates were not aggressive strains. However, the various differences between the six Israeli isolates in deduced aa in other sites of the encoded proteins (Table S1), not known to be associated with characterization of symptom phenotype, might have an effect on symptom manifestations.

For further characterization of the newly identified PepMV isolates, various factors involved in manifestations of the viral disease should be considered. Among those are the host-specific response, viral systemic spread efficiency, viral titer and environmental conditions. Regarding the host plant response, the data presented in the current study involved viral infections of the most commonly occurring commercially available tomato plants harboring the *Tm−2^2^* resistance allele, which was effective against the tobamoviruses tobacco mosaic virus (TMV) and tomato mosaic virus (ToMV) but has been broken by ToBRFV [2]. Regarding viral titers and spread in correlation with environmental conditions, PepMV isolates identified as CH2 strains are highly responsive to temperature variations [28]. Accordingly, the new PepMV-Ya isolate CP levels showed an increase in systemic viral titer upon temperature reduction by a factor of 3.0 ± 1.7 s.d. (*n* = 4) (Figure 4), suggesting that the tested unaggressive isolate was not impaired in viral replication machinery or plant systemic spread and was responsive to an environmental temperature change. In addition, PepMV-Ya singly infected tomato plants caused viral spread in an infectivity potential assay with uninfected tomato plants, showing viability of the PepMV viral particles (Figure 3) in the mildly symptomatic singly inoculated plants.

HTS analysis of the field-collected symptomatic tomato plants showed the prevalence of ToBRFV and PepMV in the viral RNA extraction. Importantly, no other plant viruses were detected. In addition, only one PepMV strain was recovered by whole-genome sequencing of PepMV purified from field-collected tomato plants showing six distinct manifestations of the viral disease, suggesting that the observed severe symptoms could not be attributed to mixed infections with various PepMV strains [27]. Therefore, our laboratory inoculation studies, showing increased PepMV-Ya titers in ToBRFV and PepMV-Ya co-infected tomato plants compared to PepMV-Ya single infections (Figure 4, Figure 6 and Table S2), present appropriate model systems reflecting the possible causal factors involved in the severe symptomatic tomato plants collected from the field. For a more accurate characterization of the field-collected symptomatic tomato plants, the possible contributions of aa differences observed in the various encoded proteins of the six isolates to symptom manifestations need to be studied as well. Importantly, the ToBRFV and PepMV-Ya co-inoculated plants showed increased severe symptoms on plant leaves when compared to PepMV-Ya single inoculations (Figure 2, Figure 4 and Table 3) and the high PepMV-Ya titers in the co-inoculated plants were associated with SF > 1 calculated for proportional plant height reductions and an enhanced total viral load (Table 4).

Interactions between tobamoviruses and potexviruses and the contribution of tobamoviruses to increased titers of a heterologous co-infecting virus have been observed before. For example, induction of replication efficiencies of both cymbidium mosaic potexvirus and odontoglossum ring spot tobamovirus in mixed-infected protoplasts has been reported [48]. In addition, an effect of tobamovirus MP on complementation of potexvirus cell-to-cell impaired movement has been shown [49,50]. In tomatoes, enhancement of symptom severity and potexvirus titer have been observed in plants mixed-infected with TMV and the potexvirus potato virus X (PVX) [51,52,53], or mixed-infected with ToMV and PVX [54]. Interestingly, tobamovirus and potyvirus mixed-infected *Solanum brevidens* plants showed a high titer of the potyvirus although most mixed infections with potyviruses involved steady unaffected levels of the potyvirus and increased levels of the heterologous co-infecting viruses [55]. These data strengthen our results regarding the effect of ToBRFV on enhanced PepMV expression in the co-infected tomato plants. Most interesting are reports regarding manifestations of viral synergies involving potexviruses, showing maximal effects on the potexvirus accumulation when potexvirus infections were second in order [56,57]. Our results showing a prominent effect of ToBRFV on PepMV accumulations in tomato plants pre-inoculated with ToBRFV seem to be in line with those reports (Figure 6 and Table S2).

The mechanism involved in ToBRFV effect on PepMV titers in the mixed-infected tomatoes could be the activity of RNA silencing suppression attributed to 126-kDa and 130-kDa small RdRp subunits of the tobamoviruses: TMV and ToMV, respectively [58,59,60]. RNA silencing is a major plant response to viral infection, suppressing viral gene expression post-transcriptionally [61,62]. While counteracting this plant response, many viral RNA silencing suppressors (VSRs) promote viral genome amplification and virus spread through increased cell-to-cell and long distance movement [63]. Enhancement of potexvirus viral titers and pathogenicity involving expression of VSRs from a heterologous virus has been observed [64]. In addition, the involvement of plant RNA silencing in potexvirus long distance movement and the contribution of the potexvirus VSR to viral cell-to-cell movement have been shown [65,66], strengthening the possible contribution of tobamovirus VSRs to increased PepMV systemic titers.

The severe tomato plant symptom manifestations occurring in the ToBRFV and PepMV mixed-infected tomato plants could be associated with VSR activities and/or the PepMV high titers. VSR interference with the plant microRNA pathways, which participate in regulation of plant growth and development, has been a suggested mechanism for enhanced symptom development associated with synergy between a potexvirus and a potyvirus [67]. Regarding PepMV high titer effect on symptom development, a mild PepMV isolate accumulation in *Nicotiana benthamiana* expressing p19 RNA silencing suppressor was correlated with a necrotic phenotype induction, which was attributed to the conserved GDD triplet motif at the polymerase aa domain of the RdRp (Figure 1) [28,68]. Interestingly, the shoestring-like leaf symptom, observed in tobamovirus infected tomato plants [69,70], was augmented in the ToBRFV and PepMV mixed-infected plants compared to ToBRFV single infections (Figure 2), suggesting that features of tobamovirus biological effects were also affected by the mixed-infection conditions. Whether the observed ToBRFV effects on PepMV titers and/or symptom phenotypes in mixed-infected tomato plants involved VSR activities or direct interactions between the viruses [71] is an intriguing question that needs further molecular analyses of this synergism.

## 5. Conclusions

The tobamovirus ToBRFV, a major disease-causing agent in tomato plants, could evidently initiate distinctly severe symptoms in the plants upon co-infections with a mild potexvirus, the Israeli PepMV CH2 isolate. Interestingly, the severe symptoms were characteristic of the effects a PepMV aggressive strain, indicating the crucial role of PepMV in disease manifestations. The severe disease was associated with high PepMV titers, observed more profoundly when ToBRFV preceded PepMV in the sequential inoculation experiments. ToBRFV could therefore be involved in enhancement of PepMV titers in the mixed-infected plants. This deduced conclusion could serve tomato growers in combating the new severe disease. Implementing current approaches to prevent ToBRFV disease spread by breeding new ToBRFV-resistant tomato varieties as well as inducing cross protection with mild tobamovirus species or strains would be an important strategy towards neutralizing the ToBRFV effects on PepMV in mixed infections.

## Figures and Tables

**Table 1:** Primer pairs served for tomato brown rugose fruit virus (ToBRFV) and pepino mosaic virus (PepMV) detection and PepMV whole-genome sequencing.

Set No.	Orientation	Name (nt. Position)	Amplicon Length (bp)	Sequence (5′-3′)
1	F	ToBRFV (5557)	615	TTTAGTAGTAAAAGTGAGAAT
	RC	ToBRFV (6167)		TTGTAAACCGGATGCACTTTCAAATG
2	F	PepMV (5658)	650	CCATCAGATGCACCACCAAC
	RC	PepMV (6307)		TTAGCTCCTCCCATGTGTCC
3	F	PepMV (1)	1184	GAAAACAAAACATAACACATAATA
	RC	PepMV (1184)		AAAAACTTGTCGCACCCATG
4	F	PepMV (925)	1070	AACATCTTCCATCCCCAACA
	RC	PepMV (1994)		CGTCTTCTCTGCCATGTGAA
5	F	PepMV (1711)	1164	TGGGCTAGTCTTGCATCTGA
	RC	PepMV (2874)		GTCTCGTGCAGTTGATTCCA
6	F	PepMV (2698)	1205	CAAAAGTGCAAAGTGCCAAT
	RC	PepMV (3902)		CTGGTGAAGGTCCCACATTT
7	F	PepMV (3619)	1097	TTAAGGAAAATGCGGCAAAG
	RC	PepMV (4715)		ATTTTTGGTGACCCCTGTCA
8	F	PepMV (4522)	1130	AACTGGGAAAACCACATTGC
	RC	PepMV (5651)		CTAACCCATCAGATGCACCA
9	F	PepMV (6075)	340	GCAAAATTGGGCTATCAGGA
	RC	PepMV (6414)		ATTTAGTAGATTTAGATACTAAGG

F, forward; RC, reverse complement; nt, nucleotide.

**Table 2:** Viral analyses of tomato plants collected from 104 greenhouse tomato plots in Israel, using enzyme-linked immunosorbent assay (ELISA) and RT-PCR.

Location (Number of Samples)	ELISA		^a^ RT-PCR	
	ToBRFV	PepMV	ToBRFV	PepMV
1. Ramat Negev (11)	11/11	10/11	11	10
2. Bsor (69)	69/69	50/69	69	50
3. Lakhish (5)	5/5	5/5	5	3
4. Arava (5)	5/5	5/5	5	2
5. Beit She’an Valley (8)	5/8	5/8	5	1
6. Hefer Valley (6)	6/6	6/6	6	6

^a^ RT-PCR validations of selected ELISA-positive samples; ToBRFV, tomato brown rugose fruit virus; PepMV, pepino mosaic virus.

**Table 3:** Symptom manifestations in mixed-infected tomato plants inoculated with a mixture of tomato brown rugose fruit virus (ToBRFV) and pepino mosaic virus (PepMV) or sequentially by pre-inoculations with PepMV or ToBRFV.

Temp.	PepMV	ToBRFV	Mixed	PepMV »ToBRFV	ToBRFV »PepMV
18 °C	LY (20–30%) LM (20–30%)	LY (20–30%) LM (80–90%) LD (20–30%)	LY (20–30%) LM (80–90%) LD (80–90%)	LY (60–70%) LM (60–70%) LD (60–70%)	LY (80–90%) LM (80–90%) LD (80–90%)
25 °C	NS	LM (60–70%) LD (20–30%)	LY (60–70%) LM (60–70%) LD (60–70%)	LY (20–30%) LM (80–90%) LD (60–70%)	LY (60–70%) LM (60–70%) LD (20–30%)
32 °C	NS	LM (20–30%) LD (60–70%)	LM (60–70%) LD (20–30%)	LY (60–70%) LM (60–70%) LD (60–70%)	LY (20–30%) LM (20–30%) LD (60–70%)

Temp., temperature; LY, leaf yellowing; LM, leaf mosaic; LD, leaf deformations; NS, no symptoms; in brackets, estimated percentages of phenotype degree of severity.

**Table 4:** Synergy factor of tomato brown rugose fruit virus (ToBRFV) and pepino mosaic virus (PepMV) co-inoculation effects on tomato plant heights and total viral load.

**Treatment**	**Temp.**	**Mean Heights (cm)**	**s.d. (*n* = 4)**	**% Height Reductions**		**SF**
Healthy	18	77	7.39	0%		-
Mixed	18	46.75	2.5	39%		1.10
PepMV»ToBRFV	18	43.25	2.22	44%		1.23
PepMV	18	52.5	2.38	32%		-
ToBRFV	18	74	1.63	4%		-
**Treatment**	**Temp.**	**Mean Number of Leaves**	**s.d.**	**% Leaf Reductions**		**SF**
Healthy	18	10.75	0.5	0%		-
Mixed	18	10	0.82	7%		2.99
PepMV»ToBRFV	18	9	0.82	16%		6.97
PepMV	18	9.5	1.29	12%		-
ToBRFV	18	11.75	1.5	−9%		-
**Treatment**	**Temp.**	**Mean Intensity Ratios, PepMV**	**s.d.**	**Mean Intensity Ratios ToBRFV**	**s.d.**	**SF**
PepMV	18 °C	76%	35%	-	-	-
ToBRFV	18 °C	-	-	64%	49%	-
PepMV»ToBRFV	18 °C	125%	33%	120%	42%	1.75
ToBRFV»PepMV	18 °C	190%	106%	250%	95%	3.14
PepMV	25–45 °C	20%	3%	-	-	-
ToBRFV	25–45 °C	-	-	117%	37%	-
Mixed	25–45 °C	123%	31%	78%	18%	1.47

Temp., temperature; s.d., standard deviation of the mean; SF, synergy factor.

## Supplementary Materials

The following are available online at https://www.mdpi.com/1999-4915/12/8/879/s1. Table S1: Differential deduced amino acids in six Israeli pepino mosaic virus isolates. Table S2: Relative gene expressions and relative gene expression ratios of tomato brown rugose fruit virus (ToBRFV) and pepino mosaic virus (PepMV), normalized to tomato endogenous gene *TIP41* in mixed-infected tomato plants inoculated with a mixture of the viruses or sequentially, by pre-inoculations with PepMV or ToBRFV.
