# Supplementary material for: Tomato Brown Rugose Fruit Virus Contributes to Enhanced Pepino Mosaic Virus Titers in Tomato Plants
Source: Viruses. 2020 Aug 11;12(8):879. doi: 10.3390/v12080879 (PMC7472245; doi:10.3390/v12080879)
Supplement: Supplementary file 1 [file viruses-12-00879-s001.pdf]

Supplementary Files

Table S1. Differential deduced amino acids in six Israeli pepino mosaic virus isolates.

#	Gene	Position aa.	Position bp.	Ya		Mi		RN		Mo		Ah		Ye	
				aa	nt.	aa	nt.	aa	nt.	aa	nt.	aa	nt.	aa	nt.
1	RdRP	10	29	K	AAG	K	AAG	K	AAG	K	AAG	R	AGG	K	AAG
2	RdRP	140	419	S	AGC	N	AAC	N	AAC	S	AGC	S	AGC	S	AGC
3	RdRP	182	544	I	ATC	I	ATC	I	ATC	I	ATC	V	GTC	I	ATC
4	RdRP	328	982	A	GCT	A	GCT	A	GCT	S	TCT	A	GCT	A	GCT
5	RdRP	346	1,036	A	GCA	A	GCA	A	GCA	T	ACA	T	ACA	T	ACA
6	RdRP	379	1,135	C	TGC	C	TGC	C	TGC	C	TGC	R	CGC	C	TGC
7	RdRP	426	1,276	T	ACT	A	GCT	A	GCT	A	GCT	A	GCT	A	GCT
8	RdRP	466	1,397	S	TCT	F	TTT	F	TTT	F	TTT	F	TTT	F	TTT
9	RdRP	468	1,402	S	TCC	S	TCC	S	TCC	P	CCC	S	TCC	S	TCC
10	RdRP	474	1,421	R	CGG	R	CGG	R	CGG	R	CGG	Q	CAG	R	CGG
11	RdRP	486	1,456	K	AAG	K	AAG	E	GAG	K	AAG	K	AAG	K	AAG
12	RdRP	488	1,462	N	AAT	N	AAT	D	GAT	N	AAT	N	AAT	N	AAT
13	RdRP	1,085	3,254	A	GCG	V	GTG	A	GCG	A	GCG	A	GCG	A	GCG
14	RdRP	1,090	3,268	A	GCC	A	GCC	A	GCC	T	ACC	A	GCC	A	GCC
15	RdRP	1,132	3,394	S	TCT	S	TCT	S	TCT	P	CCT	S	TCT	S	TCT
16	RdRP	1,287	3,859	I	AAT	I	AAT	I	AAT	I	AAT	V	GTT	I	AAT
17	RdRP	1,365	4,093	H	CAT	H	CAT	H	CAT	H	CAT	Y	TAT	H	CAT
18	RdRP	1,390	4,168	H	CAT	H	CAT	H	CAT	H	CAT	Y	TAT	H	CAT
19	TGB1	125	373	Q	CAA	K	AAA	K	AAA	K	AAA	K	AAA	K	AAA
20	TGB1	136	406	N	AAT	D	GAT	D	GAT	D	GAT	D	GAT	D	GAT
21	TGB1	232	696	S	TCT	F	TTT	S	TCT	S	TCT	S	TCT	S	TCT
22	TGB3	61	181	N	AAC	N	AAC	N	AAC	N	AAC	D	GAC	N	AAC
23	CP	12	34	N	AAT	D	GAT	D	GAT	D	GAT	D	GAT	D	GAT
24	CP	50	148	A	GCT	T	ACT	T	ACT	T	ACT	T	ACT	T	ACT

aa, amino acid; bp, base pairs; nt, nucleotide.

Table S2. Relative gene expressions and relative gene expression ratios of tomato brown rugose fruit virus (ToBRFV) and pepino mosaic virus (PepMV), normalized to tomato endogenous gene *TIP41*, in mixed infected tomato plants inoculated with a mixture of the viruses or sequentially, by pre-inoculations with PepMV or ToBRFV.

Sample Name PepMV 18°C.	Mean ΔC_T	Mean $\Delta\Delta C_T$	s.d. (n)	p-Values	2^-$\Delta\Delta C_T$	s.d. 2^-$\Delta\Delta C_T$
Single	-11.78	0	- (6)	x	1	-
Mixed	-13.58	-1.80	2.00 (6)	1.75E-05	20.37	18.00
PepMV»ToBRFV	-15.80	-4.02	1.83 (8)	0.042	5.19	5.47
ToBRFV»PepMV	-15.28	-3.49	3.65 (8)	3.28E-04	19.60	10.56

Sample Name ToBRFV 18°C	Mean ΔC_T	Mean $\Delta\Delta C_T$	s.d. (n)	p-Values	2^-$\Delta\Delta C_T$	s.d. 2^-$\Delta\Delta C_T$
Single	-6.49	0	- (5)	x	1	-
Mixed	-5.57	0.92	3.02 (6)	0.67	3.01	5.29
PepMV»ToBRFV	-7.40	-0.91	1.55 (8)	0.38	1.69	2.22
ToBRFV»PepMV	-5.94	0.55	4.14 (8)	0.02	2.25	1.58
**Sample Name PepMV 25°C**	**Mean ΔC_T**	**Mean $\Delta\Delta C_T$**	**s.d. (n)**	**p-Values**	**2^-$\Delta\Delta C_T$**	**s.d. 2^-$\Delta\Delta C_T$**
Single	-7.66	0	- (6)	x	1	-
Mixed	-14.21	-6.55	1.57 (8)	1.55E-03	105.11	50.32
PepMV»ToBRFV	-14.30	-6.64	1.77 (7)	2.19E-05	135.61	78.03
ToBRFV»PepMV	-13.94	-6.28	1.29 (7)	4.03E-03	95.99	63.96
**Sample Name ToBRFV 25°C**	**Mean ΔC_T**	**Mean $\Delta\Delta C_T$**	**s.d. (n)**	**p-Values**	**2^-$\Delta\Delta C_T$**	**s.d. 2^-$\Delta\Delta C_T$**
Single	-6.90	0	- (8)	x	1	-
Mixed	-6.18	0.72	1.54 (6)	2.30E-03	6.88	3.49
PepMV»ToBRFV	-6.16	0.73	1.80 (8)	0.37	0.74	0.51
ToBRFV»PepMV	-9.48	-2.59	1.45 (8)	0.54	0.89	0.85
**Sample Name PepMV 32°C**	**Mean ΔC_T**	**Mean $\Delta\Delta C_T$**	**s.d. (n)**	**p-Values**	**2^-$\Delta\Delta C_T$**	**s.d. 2^-$\Delta\Delta C_T$**
Single	-9.94	0	- (6)	x	1	-
Mixed	-10.63	-0.69	2.63 (8)	9.86E-06	21.55	16.92
PepMV»ToBRFV	-13.52	-3.58	1.86 (8)	0.04	1.97	1.34
ToBRFV»PepMV	-13.98	-4.03	2.06 (8)	4.54E-05	13.49	6.70
**Sample Name ToBRFV 32°C**	**Mean ΔC_T**	**Mean $\Delta\Delta C_T$**	**s.d. (n)**	**p-Values**	**2^-$\Delta\Delta C_T$**	**s.d. 2^-$\Delta\Delta C_T$**
Single	-7.23	0	- (6)	x	1	-
Mixed	-5.96	1.27	1.20 (6)	0.78	1.10	0.32
PepMV»ToBRFV	-6.01	1.23	1.44 (8)	0.01	0.50	0.31
ToBRFV»PepMV	-7.32	-0.09	1.30 (8)	0.04	0.60	0.48

s.d., standard deviation of the mean; p-Values, obtained by t-Test: Two sample assuming unequal variances.
